# Endophytic Fungi-Mediated Biocatalysis and Biotransformations Paving the Way Toward Green Chemistry

**DOI:** 10.3389/fbioe.2021.664705

**Published:** 2021-06-16

**Authors:** Malvi Choudhary, Suruchi Gupta, Manoj K. Dhar, Sanjana Kaul

**Affiliations:** School of Biotechnology, University of Jammu, Jammu, India

**Keywords:** bioactive metabolites, biocatalyst, biotechnological applications, biotransformation, endophytes, genetic diversity

## Abstract

Catalysis is a process carried out in the presence of a heterogenous catalyst for accelerating the rate of a chemical reaction. It plays a pivotal role in transition from take, make, and dispose technology to sustainable technology via chemo- and biocatalytic processes. However, chemocatalyzed reactions are usually associated with copious amounts of perilous/hazardous environmental footprints. Therefore, whole-cell biotransformations or enzyme cocktails serve as cleaner biocatalytic alternatives in replacing the classical chemical procedures. These benchmark bioconversion reactions serve as important key technology in achieving the goals of green chemistry by eliminating waste generation at source. For this, nature has always been a driving force in fuelling natural product discovery and related applications. The fungal endophytic community, in particular, has undergone co-evolution with their host plant and has emerged as a powerful tool of genetic diversity. They can serve as a treasure trove of biocatalysts, catalyzing organic transformations of a wide range of substances into enantiopure compounds with biotechnological relevance. Additionally, the biocatalytic potential of endophytic fungi as whole-intact organisms/isolated enzyme systems has been greatly expanded beyond the existing boundaries with the advancement in high-throughput screening, molecular biology techniques, metabolic engineering, and protein engineering. Therefore, the present review illustrates the promising applications of endophytic fungi as biocatalysts for the synthesis of new structural analogs and pharmaceutical intermediates and refinement of existing proteins for novel chemistries.

## Introduction

**FIGURE 1:**
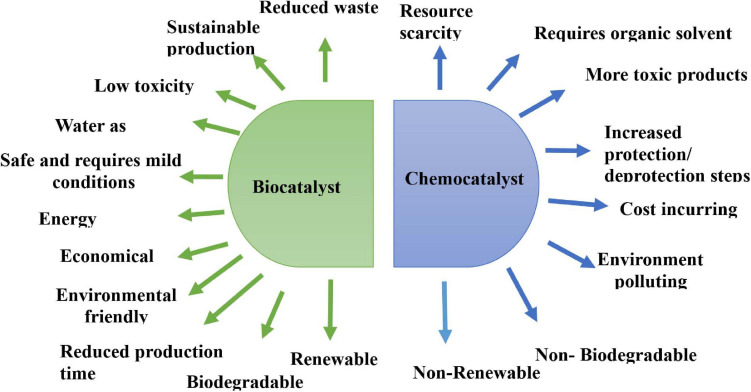
Advantages of biocatalyst over chemocatalyst.

The transformation process is defined as the conversion of exogenous substances into new chemical moieties. It is achieved by introducing the structural modifications in the original carbon skeleton of substrates. Catalysts are generally employed to enhance the rate of transformation processes without being consumed during the reaction. Such a process involving the use of a catalyst in accelerating the rate of a chemical reaction is termed as catalysis (Sheldon and Woodley, 2018). It can be categorized as chemocatalysis and biocatalysis. Chemocatalysis is defined as the organic reaction that operates under extreme conditions, utilizes low atom economy, and generates toxic and hazardous waste stream, thereby impacting the environment adversely (Kim and Li, 2020). Chemical synthesis poses many distinct challenges and shortfalls like the utilization of organic solvents, multistep reactions, the need for protection/deprotection loops, requirement for transition metal cofactors, and toxic reaction products (Schwartz et al., 2014). Such an increasing complexity of industrial pipelines can be circumvented by surpassing the thermodynamic hurdles via microbial cascade biotransformations. In this scenario, the introduction of biocatalysis is the revolutionary implementation of microbial biotransformation potential in the field of catalysis. Biocatalysis is a process to repurpose the enantio- and regioselectivity of existing molecules for enhanced novel chemistries. This can be envisioned by whole intact organisms or isolated enzymes for the creation of value-added products by minimizing the industrial non-selective hazardous chemicals and waste. Furthermore, the noteworthy pros and cons of both transformation reactions are highlighted in Figure 1. The ongoing trend focuses on the conservation of the Earth’s natural resources, maintenance of life quality, reduction of industrial activities, and efficient use of renewable raw materials for the successful translation and implementation of chemistry and biotechnology into sustainable technology (Truppo, 2017). Also, the ever-rising demand of new medicines for managing a constellation of human maladies has expedited the research on microbial diversity as an underexplored group of microorganisms for providing novel enzymes in transforming chemical entities (Kinch et al., 2020).

**FIGURE 2:**
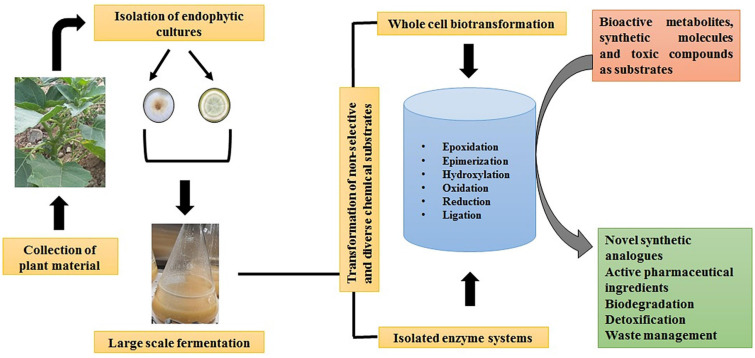
A schematic representation of the biotransformation process mediated by fungal endophytes.

In this regard, the endophytic fungal community having undergone million of years of directed evolution has come up as new drivers in drug discovery space by producing a plethora of bioactive natural products (Rodriguez et al., 2016). Therefore, the biotransformation reactions mediated by endophytic fungi/isolated enzyme systems are gaining tremendous attention among process chemists for sustainable pharmaceutical development (Figure 2). Therefore, the present review highlights the multifaceted potential of endophytic fungi-assisted biotransformations in the bioremediation of hydrocarbons, toxic heavy metals, decolorization of dyes, and detoxification of xenobiotic pollutants, besides the enzymatic and scalable transformation of non-selective and diverse chemical substrates into active pharmaceutical ingredients.

## Endophytes as Advantageous Biocatalyst Over Conventional Catalyst

Conventional catalysts are bestowed with catalytic efficiency in transforming substrates to specific products by lowering the free energy of the transition state. In the past decades, they have been featured as a complementary tool for organic conversions especially in organic chemistry and pharmaceutical industry. However, chemical synthesis based on conventional catalysts pose many inherent drawbacks like cost incurrence, requirement for specially designed equipment, control systems, complicated refining and purification, unwanted by-products, and enhanced environmental pollution. These traditional chemical transformations have raised many public and ecological concerns in terms of high capital investment, high energy cost, and hazardous waste products. In this regard, microbe-assisted transformation is considered as one of the luring technological solutions to mitigate the drawbacks posed by these conventional catalysts. Among the microbes, fungal endophytes and its enzymatic cascade are now becoming a key component of toolbox assisting in the process chemistry/synthetic processes for the production of molecules of industrial interest. Herbaceous plants have co-evolved with species-rich fungal endosymbionts that are known to inhabit every accessible plant organ starting from rhizosphere to phylloplane, caulosphere, anthrosphere, carposphere, and finally spermosphere (Khan et al., 2017; Yan et al., 2018). Fungal endophytes comprised of horizontally transmitted ecological group of asymptomatic microorganisms imparting beneficial attributes to the host plant (Gange et al., 2019). They establish universal relationships with the plant species ranging from symbiosis to latent pathogens. This plant–endophyte interaction is intricately modulated by environmental parameters, host genotype, developmental stage, and immune system signaling (Aamir et al., 2020). Unveiling the folds of endophyte–host interaction reveals a hotspot for the exploration of repertoire of hyperdiverse and valuable secondary metabolites.

In the current milieu, most of the reports are focused on the richness and biological perspective of endophytic fungal metabolites. However, among the essential biochemical resources, extracellular hydrolase production outstands for their immediate implications in the realms of medicine, agriculture, and industry. Since, endophytic fungal enzymes such as pectinases, cellulases, and lipases play an important role in breaching the plant barriers for the establishment of symbiotic association by counteracting invading plant pathogens and obtaining nutrients from the host plant (Vasundhara et al., 2019). Therefore, endophytic fungal lipases, amylases, tyrosinases, proteases, phosphatases, and L-asparaginases have prodigious effects in lignocellulosic biomass degradation, processing of raw materials, bioremediation, fermentation, and many industrial applications (Rana et al., 2019). In addition, enhanced chemo-, regio-, and stereoselective conversion of synthetic molecules with reduced consumption of organic solvents and waste-producing steps has resulted in the bioprospection of fungal endophytes as a source of novel biocatalyst in the green chemistry framework (Rodriguez et al., 2016). Several workers have comprehensively acknowledged the potential of underexplored endophytic fungal enzymes in industrial processes over other secondary metabolites (Corrêa et al., 2014; Khan et al., 2017; Yadav, 2019). Such a versatility in catalytic repertoire represents an unexplored inventory of novel global biocatalyst entrancing solutions to major addressed challenges in the field of health, environment, and energy production (Suryanarayanan et al., 2012; Gupta et al., 2016).

Therefore, biotransformation reactions mediated by endophytic fungi are characterized under “green biocatalysis” technology that results in environmental-friendly degradation of contaminants.

## Biotransformation Reactions Mediated by Endophytic Fungi

Biotransformation can be simply defined as a process involving a biological system in transforming the chemical compounds into new structural analogs (Figure 2). It is an enantiospecific process that modifies functional and structural moieties of organic compounds into new chiral centers (Smitha et al., 2017). It is an effective path toward white biotechnology to generate novel structural analogs or to refine the pharmacokinetic properties of natural compounds. The innovative enzyme systems of fungal endophytes as whole-intact organism or as single-enzyme system are capable of transforming several organic compounds (Bianchini et al., 2015). Single-step catalysis involving isolated enzyme are capable of catalyzing enantio- and stereoselective conversion of molecules under ambient conditions, while whole cells mediating biotransformation are useful in multi-step catalysis, eliminating the need for cofactor requirement and providing native environment, optimal enzyme concentrations, and energy status.

Therefore, whole-cell catalysis has received noteworthy attention due to the elimination of the need for enzyme isolation and purification steps that may lead to the loss of activity by disrupting native protein structure and is hence preferred over cell-free system (Rudroff et al., 2018). The scope of fungal endophytes as whole-cell biotransformation can further be expanded for generating alternative sources of limited biologically active natural compounds. This was exemplified by the work of Majeed et al. (2019) who suggested the novel bioconversion route for the production of calebin-A from curcumin assisted by endophytic fungus, *Ovatospora brasiliensis* EPE-10 MTCC 25236, isolated from the rhizomes of *Curcuma caesia*. In contrast to whole-cell biotransformation, isolated enzymes have more potential in single-step catalysis than conventional chemical approaches. The use of enzymes as a source of biocatalysis has been highlighted in the work of Zhang et al. (2020). They purified and characterized a novel β-glucuronidase (cg-GUS) from endophytic fungus *Chaetomium globosum* DX-TH53 for biocatalyzing the transformation of glycyrrhizin (GL) into glycyrrhetinic acid monoglucuronide (GAMG). GAMG is a functional sweetener with profound applications in biopharmaceutical and biotechnological industry. Therefore, the biotransformation ability of endophytic fungi has been overlooked as promising technology in terms of reusability, stability, broad substrate specificity, recovery, recyclability, and better product quality.

## Applications of Endophytic Fungi as Biocatalyst

Endophytic fungi are bestowed with inherent property of mimicking host metabolic pathways for the analogous production of bioactive molecules (Meshram and Gupta, 2019). Therefore, endophytic fungi lend novel dimensions in the discovery and development of drug programs, besides contributing significantly to clean, greener, and sustainable technologies (Sethi et al., 2017; Adams et al., 2019). Some applications of biotransformation procedures mediated by fungal endophytes vis-a-vis synthesis of novel structural analogs and active pharmaceutical ingredients, production of high-value products in aroma and perfume industry, enhancing agricultural productivity, bioconversion of monoacylglycerols, and biodegradation of hydrocarbons have been presented in the subsequent section.

### Synthesis of Novel Structural Analogs and Active Pharmaceutical Ingredients

The attractiveness of the “enzymatic one-pot reaction” concept is the most interesting inspiration from nature for pharmaceutical and fine chemical production. The development of valuable biocompounds by microorganisms as biocatalysts is the much awaited goal of synthetic chemistry (Liu et al., 2020). The biotransformation processes mediated by endophytic fungi as enzymes or as whole-cell catalysis result into new pharmaceutically active molecules. Literature reports have supported the remarkable and transferable biochemical potential of endophytic fungi into productive sectors (Santos and Silva, 2019; Fryszkowska and Devine, 2020).

For instance, Özçınar et al. (2018a) for the first time reported the potential of endophytic fungus *Alternaria eureka* in the biotransformation of bioactive sapogenin, neoruscogenin. The endophyte was capable of modifying the steroidal framework by oxidation, oxygenation, and epoxidation reactions, thereby generating fourteen new biotransformation products as confirmed by NMR and HRESIMS data analyses. Besides enhancement of the bioactive potential, whole-cell biotransformation is known to impart chemical diversification to the prevailing chemical scaffolds. In this context, the endophyte *Xylaria feejeensis* GM06 has been reported to mediate biotransformation of achiral β-mangostin into two new chiral heterocyclic scaffolds. This bioconversion resulted in the generation of two new pairs of enantiomers, mangostafeejin A and mangostafeejin B. The resulting heterocyclic scaffold has expanded the arsenal of chemically diverse bioactive compounds (Arunrattiyakorn et al., 2018).

Furthermore, the use of fungal endophytes as a whole-cell biotransformation is an emerging field of biotechnology that yields new modified compounds with increased biological activities. For example, Ekiz et al. (2018) designed experiments to study endophytic fungal utilization in biotransformation of cyclocanthogenol. Cyclocanthogenols are prominent immunomodulatory glycosides of the *Astragalus* genus, highly valued as telomerase activators in immune-related complaints or malignancies. The systematic screening studies revealed the isolation of eight chemically diverse compounds biotransformed by endophytic fungus, *Alternaria eureka* 1E1BL1. The study concluded that the endophytic fungus was capable of structurally transforming the cycloartane nucleus via hydroxylation, epoxidation, oxidation O-methylation, methyl migration, and ring-expansion reactions. Similarly, another endophytic fungus, *Camarosporium laburnicola*, isolated from *Astragalus* species has been reported in the biotransformation of *Astragalus* sapogenins. The whole-cell biotransformation reaction mediated by endophytic fungus resulted into the production of cycloastragenol derivatives with enhanced telomerase activity (Küçüksolak et al., 2019).

Also, the global demand for by-products has steadily gained momentum in the current prevailing conditions of pandemic outbreak for the conversion of macromolecules into biomedicine. Singh et al. (2019) reported the potential of cell culture of endophytic fungus *Trichothecium roseum* CIMAPN in transforming a pharmaceutical molecule, artemisinic acid, into a better derivative (3-oxoartemisinic acid) with anti-candidal potential.

Highly oxygenated schitriterpenoids are another class of specialized molecules valued for their bioactivities, but the stereo-chemical complexity associated with these molecules hurdled the chemical synthesis. Qin et al. (2019) reported for the first time the application of microbial technology in enhancing the production of schitriterpenoids. The endophytic fungus *Penicillium* sp. SWUKD4.1850 associated symbiotically with *Kadsura angustifolia* was capable of fermenting *K. angustifolia*, resulting in the production of nine new triterpenoids, kadhenrischinins A-H and 7β-schinalactone, for the first time. Similarly, biotransformation studies on endophyte *Penicillium sp. F5*, isolated from the samples of *Polygonum cuspidatum*, was reported by Xu Z. et al. (2020). The bioactive molecule, pterostilbene, was isolated as the main product from the biotransformation of resveratrol by the fungal endophyte.

Furthermore, for investigating the potential of endophytic fungi as a biocatalyst, Zhan et al. (2019) studied the biotransformation of huperzine B, a lycodium bioactive alkaloid reported from medicinal plant *Huperzia serrata* (Thunb.). It is a promising pharmaceutical ingredient for developing acetylcholinesterase (AChE) reversible inhibitors in the treatment of Alzheimer’s disease. The biotransformation of huperzine B by *Bjerkandera adusta* CCTCCM2017159, a fungal endophyte of the host plant, resulted in the isolation of novel compound 8α, 15α-epoxyhuperzine B. Also, two known huperzine B analogs, carinatumin B and 16-hydroxyhuperzine B, were also furnished by spectroscopic analysis. They reported that the fungal enzymes targeted the rigid ring structure of huperzine B and chemo-enzymatically transformed its structure by hydroxylation and epoxidation reactions. Similar biotransformation reactions of huperzine A mediated by fungal endophyte, *Irpex lacteus* CCTCCM 2017161, of the host plant was studied by Ying et al. (2019). Also, the biocatalytic efficiency of *Penicillium brasilianum* in generating active pharmacophores of gentiopicroside and swertiamarin, belonging to iridoid glycosides, has been reported by Xu Z. et al. (2020). Recently, the endophytic fungus *Penicillium oxalicum* B4 has been employed in the biotransformation of artemisinic acid to eight metabolites. The transformed metabolites, namely, 3α,14-dihydroxyartemisinic acid; 15-hydroxy-3-oxo-artemisinic acid; and 1,2,3,6-tetradehydro-12, 15-artemisindioic acid presented stronger cytotoxic and anti-inflammatory potential than the parent moiety (Tian et al., 2021).

Moreover, the utilization of fungi as an efficient, reliable, and sustainable source of enzyme systems or as whole cell in the catalysis of steroid biomolecules is the most unparalleled innovation in the discovery of drug candidates (Cano-Flores et al., 2020). In one of the reports, Özçinar et al. (2018b) studied the vital role of endophytic fungi in the generation of new oxygenated derivatives from steroidal molecules. The transformation of steroidal neoruscogenin by endophytic fungus *Neosartorya hiratsukae* yielded three hydroxylated novel compounds as confirmed by a spectral analysis. The metabolites yielded were mainly P450 monooxygenase products. Therefore, the study embarks the enzyme system of endophytes as potential biocatalyst in drug discovery program.

In addition to this, the significance of microorganisms in the functional transformation of testosterone and progestational hormones in producing an array of therapeutic modalities has been postulated by many natural product research groups (Ghasemi et al., 2014; Ahmad et al., 2016; Dong et al., 2016). The fungi are capable of performing controlled enzymatic processes for the biotransformation of steroidal hormones. One such study was conducted by Yildirim et al. (2019), reporting the hydroxylation of testosterone by *Cladosporium sphaerospermum* MRC 70266, a common endophyte of plants. Incubation of the molecule with *Cladosporium sphaerospermum* MRC 70266 started a series of reactions in metabolizing this steroid molecule into two new molecules (6b,16b,17b-trihydroxyandrost-4en-3-one6 and 6b,12b,17b-trihydroxyandrost-4-en-3-one7) and four known compounds. In contrast, *Plantago lanceolata* L. associated fungi were assessed for their applicability in bioconversion of non-steroidal anti-inflammatory drug molecules (NSAIDS). The findings highlighted that the plant-associated fungi were capable of biodegrading and modifying the NSAIDs into a less toxic form (Gonda et al., 2016). The documented reports highlighted the role of fungi in mediating the hydroxylation reactions, thus signifying the functional changes in the steroidal skeleton for the preparation of new pharmaceutical derivatives. Therefore, such biotransformation capacities reveal endophytes as an outstanding multi-enzyme system. Therefore, endophytic fungal enzyme-mediated transformation of natural metabolites provide novel avenues for generating structural diversity in chemical space.

### Production of High-Value Products in Aroma and Perfume Industry

Aroma and fragrances are highly priced natural compounds, commercially valued in pharmaceutical, food, chemical, and cosmetic industries. Since the demand of these “natural flavors” has been expanding rapidly, therefore, biotransformation and bioconversion approaches for the development of aroma compounds, natural flavors, and fragrances have started receiving more attention in industrial sector. In this regard, endophytes have been an efficient bio-route for flavor synthesis (Vespermann et al., 2017; Braga and Belo, 2019; Della Monica and Kleij, 2020). The long-term mutualistic relationship with the host has co-evolved these microbes in mimicking plant-associated metabolic pathways for generation of beneficial flavor compounds. This class of microorganisms is gaining preference for strengthening the biotechnological origin of unique aromatic compounds (Costa-Silva et al., 2020). For example, Bier et al. (2017) studied the bioconversion of terpenes by endophytic fungi for obtaining chemically diverse terpenoid derivatives. Terpenes have served as an attractive source of inspiration directed to their high functionality in the food and cosmetic industries. A preliminary study was conducted to assess the biotransformation potential of endophytic fungus, *Phomopsis* sp., isolated from *Pinus taeda* on limonene. Limonene is a cyclic monoterpene, an industrially exploited compound with favorable biological and physicochemical properties. Several biotransformed derivatives were isolated and identified such as a-terpineol, carvone, limoneno-1,2-diol, terpinen-4-ol, menthol, and carveol.

In a recent report, the biocatalytic potential of seven native endophytic fungal strains of Colombian plants was evaluated by Jaramillo et al. (2020) either by assessing *de novo* synthesis or by biotransformation efficiency. Out of seven strains, three endophytic fungi belonging to genus *Ganoderma* were found to be incredible biocatalyst for *de novo* synthesis of aroma derivatives by decolorizing β,β-carotene. Also, the strains of *Trametes elegans* showed different biotransformation performances via the oxidation of α-pinene for the production of aromatic compounds (hexahydro-3-(methylpropyl)-pyrrolo[1,2-a]pyrazine-1,4dione, methyl-3-methoxy-4H-pyran-4-one and hexahydro-3-(methylphenyl)-pyrrolo[1,2a]pyrazine-1,4-dione).

Monoterpenoids are another major component of essential oils, highly valued in pharmaceutical industry and perfume industry for its aroma, antibacterial, and insect repellent properties. The bioconversion of monoterpenoids was evidenced by the employment of endophytic fungi under eco-friendly conditions for biocatalyzing complex essential oils into active pharmaceutical ingredients (Cecati et al., 2018). The study was focused on the utilization of three endophytic fungal strains *Fusarium solani* Eb01, *Alternaria alternata* Eb03, and *Neofusicoccum* sp. Eb04 in diversifying chemical matrices of *Eupatorium buniifolium* essential oil into limonene, pinene, and sabinene. Recently, Yang and Wang (2021) studied the biotransformation capability of eleven endophytic fungal strains isolated from *Praxelis clematidea*. The endophytic strains biotransformed the substrate monoterpenoids, namely, (+)-limonene, (-)-limonene, and mycrene into limonene -1,2-diol, limonene, and linalool oxide as confirmed by chromatographic analyses. These chemical modifications mediated by endophytic fungi concluded that enzyme system of endophytes serves as benchmarks in scrutinizing the ongoing chemical processes into greener technology.

### Bioconversion of Monoacylglycerols

Endophytic fungal lipases are undoubtedly the most valuable enzyme representative class in applied biocatalysis. The wide substrate acceptability of fungal lipases makes them the nature’s most prominent class of enzymes (de María et al., 2019). A detailed literature on the industrial uses, synthesis of active chiral centers, and the granted patents have already been documented. For instance, an endophytic lipase-catalyzed process was carried out in bioconversion of monoacylglycerols into solketal 1-monoacylglycerol, an industrially important compound. The lipase enzyme was isolated from the extracts of endophytic fungi *Stemphylium lycopersici* and *Sordaria* sp., and the biocatalytic capability of their extracts was reported for the first time (Rocha et al., 2020). Recently, a lipase-immobilized derivative system onto agricultural by-products showed an enhanced esterification activity and stability. This innovative *Cercospora kikuchii* lipase immobilization protocol on eco-friendly supports was reported by Costa-Silva et al. (2020) for complementing organic synthesis. Hence, lipase-mediated biotransformation processes are a fascinating research subject for improving the chemical modifications of natural products.

### Enhancing Agricultural Productivity

Hazardous contaminants, metalloids, and metal pollutants released from industrial discharge and other human activities accumulate in the ecosystem and result in toxicity to environment and human health. Plant metal toxicity is one of the major concerns that interferes with crop production. An alternative strategy to sequester, detoxify, and extract toxic metals and xenobiotic contaminants from soil, air, or water is achieved by the process of phytoremediation (He et al., 2020). *Ex situ* remediation strategies using chemical catalysis result in the generation of wastes, cost incurrence, poor recovery, and undesirable environmental and health attributes. For detoxification of environment, the use of endophytic fungal enzymes as biocatalysts is an emerging technology for eliminating detrimental toxins from soil with enduring application (Domka et al., 2019). For example, the bioremediation potential of endophytic fungi capable of metabolizing toxic macromolecules into smaller and less toxic form has been reported by Hussain et al. (2018). They have reported the biotransformation ability of four endophytic fungal strains *Aspergillus fumigatus*, *Rhizopus* sp., *Penicillium radicum*, and *Fusarium proliferatum* capable of transforming highly toxic hexavalent form of chromium to its less toxic trivalent form, thereby promoting growth of *Lactuca sativa* in chromium toxic soil. In another study, *in vitro* bioremediation potential of endophytic fungal consortium isolated from *Agrostis stolonifera* in lead-contaminated soil has been reported to stimulate the growth of test plant *Festuca arundinacea* Schreb under heavy metal toxicity (Soldi et al., 2020). Similarly, endophytic fungus, *Trametes hirsuta*, isolated from lead-stressed *Chenopodium album* L. plant has been documented to provide lead tolerance to *Triticum aestivum* L. seedlings and thus resulted in endophyte-assisted phytoremediation (Malik et al., 2020). The recent functional role of mercury-resistant endophytic fungal strains (*Aspergillus* sp. A31, *Curvularia* geniculata P1, *Lindgomycetaceae* P87, and *Westerdykella* sp. P71) in bioremediating mercury in a species-dependent manner has been studied by Pietro-Souza et al. (2020). These endophytic strains bioaccumulate the toxic metal in plant tissues thereby resulting in mercury volatization, besides promoting the *in vitro* growth of *Aeschynomene fluminensis* and *Zea mays.* Hence, fungal endophyte-assisted biotransformation bear the “green” label as a bioremediating agent.

### Waste Management

Generation of petrochemical wastes, aliphatic and aromatic hydrocarbons, as by-products of crude oil refining industry results in ecological imbalance and toxicity due to their mutagenic and toxic properties. The involvement of microorganisms as whole cells or as isolated enzyme systems in transforming highly polluted organic contaminants into simpler substances is known as bioremediation, a clean and waste-free technology (Asemoloye et al., 2020). Since, endophytic fungi are intimately associated with plants and produce a unique plethora of secondary metabolites that have the ability to transform, degrade, and detoxify hydrocarbons into safe and environmentally friendly substances. Therefore, fungal endophytes find immense application as bioremediating agents. Mishra et al. (2019) have systematically reviewed the hydrolytic and oxidative enzymatic potential of endophytic fungal candidates in metabolizing scalable transformation of non-selective and diverse chemical substrates. Bioprospecting endophytic fungal enzymes (manganese peroxidase, lignin peroxidase, cellulase, chitinase, laccase, lipoidase, etc.) in the biodegradation of hydrocarbons such as polycyclic aromatic hydrocarbons and polychlorinated biphenyls have been reviewed in depth by Krishnamurthy and Naik (2017). Pyrolysis of these hydrocarbons by chemocatalysis usually results in the generation of mutagenic and carcinogenic intermediates that remain persistent in natural matrices. Therefore, endophytic enzymes have emerged as a promising option in bioremediation of metal-contaminated environment. Similar to this finding, endophytic fungal species belonging to the genera *Verticillium* and *Xylaria* isolated from crude oil-contaminated habitats were evaluated and reported for the first time as an efficient petroleum hydrocarbon biodegraders by Marín et al. (2018). Likewise, the efficiency of endophytes in degrading the petroleum hydrocarbons has also been reported by Sawant and Rodrigues (2020). Seven mangrove-inhabiting endophytic fungi, viz., *Nigrospora* sp., *Aspergillus niger*, *Aspergillus* sp., *Curvularia* sp., *Pestalotiopsis adusta*, *Fusarium* sp., and *Cladosporium* sp. were evaluated for degrading hydrocarbons. Among them, *Nigrospora* sp. possessed the highest hydrocarbon biodegradation ability as analyzed by FT-IR spectroscopy.

Furthermore, triphenylmethane (TPM) dyes constitute an important group of synthetic dyes that includes cotton blue, crystal violet, malachite green, and methyl violet. These synthetic dyes finds application in textile, leather, paper, cosmetic, and pharmaceutical sectors. However, their degradation procedure generates many hazardous and carcinogenic products into the environment. Gao et al. (2020) reported that the endophytic fungus *Bjerkandera adusta* inhabiting the roots of *Sinosenecio oldhamianus* was capable of decolorizing and detoxifying TPM dyes. The mechanisms responsible for biodecolorization and biodegradation were either due to biosorption or degradation by enzymes (manganese peroxidase and lignin peroxidase) as confirmed by FTIR analysis, UV-spectra, and phytotoxicity tests. Thus, an endophytic fungi-mediated *in situ* biodegradation process can be used as an alternative green technology in degrading environmental contaminants.

Furthermore, substantial examples on the biocatalytic potential of endophytic fungi in the biotransformation of molecules are tabulated in Table 1.

**TABLE 1:** Summary of recent biotransformation reactions mediated by endophytic fungi.

**S. no.**	**Fungal endophyte**	**Host plant**	**Substrate**	**Transformed products**	**References**
1.	*Alternaria eureka*	*Astragalus angustifolius*	Sapogenin, neoruscogenin	Fourteen new biotransformation products	Özçınar et al., 2018a
2.	*Xylaria feejeensis*	*Garcinia mangostana*	β-mangostin	Mangostafeejin A and mangostafeejin B	Arunrattiyakorn et al., 2018
3.	*Alternaria eureka*	*Astragalus* sp.	Cyclocanthogenol	Eight new compounds	Ekiz et al., 2018
4.	*Camarosporium laburnicola*	*Astragalus* sp.	Cyclocanthogenol	Cycloastragenol derivatives	Küçüksolak et al., 2019
5.	*Trichothecium roseum*	*Artemisia annua*	Artemisinic acid	3-oxoartemisinic acid	Singh et al., 2019
6.	*Penicillium* sp.	*Kadsura angustifolia*	Schitriterpenoids	Kadhenrischinins A–H and 7β-schinalactone	Qin et al., 2019
7.	*Penicillium* sp.	*Polygonum cuspidatum*	Resveratrol	Pterostilbene	Xu Z. et al., 2020
8.	*Bjerkandera adusta*	*Huperzia serrata*	Huperzine B	8α, 15α−epoxyhuperzine B, carinatumin B, 16−hydroxyhuperzine B	Zhan et al., 2019
9.	*Irpex lacteus*	*Huperzia serrata*	Huperzine A	8α,15α-epoxyhuperzine A	Ying et al., 2019
10.	*Penicillium brasilianum*	*Gentiana rigescens*	Gentiopicroside and swertiamarin	Seven products, including one new compound, 5−ethylidene−8−hydroxy−4,5,6,8− tetrahydropyrano[3,4−c]pyran−1−one	Xu L.L. et al., 2020
11.	*Penicillium oxalicum*	*Artemisia annua*	Artemisinic acid	3α,14-dihydroxyartemisinic acid; 15-hydroxy-3-oxo-artemisinic acid; and 1,2,3,6-tetradehydro-12, 15-artemisindioic acid	Tian et al., 2021
12.	*Neosartorya hiratsukae*		Neoruscogenin	Three hydroxylated novel compounds	Özçinar et al., 2018b
13.	*Cladosporium sphaerospermum*		Testosterone	6b,16b,17b-trihydroxyandrost-4en-3-one6 and 6b,12b,17b-trihydroxyandrost-4-en-3-one7	Yildirim et al., 2019
14.	*Alternaria alternata* and *Trichoderma* sp.	Medicinal and aromatic plants	Rose oil distillation wastewater	Phenolic compounds	Rusanova et al., 2019
15.	*Ganoderma* sp. and *Trametes elegans*		Decolorizing β,β-carotene and oxidation of α-pinene	Aroma derivatives (hexahydro-3-(methylpropyl)-pyrrolo[1,2-a]pyrazine-1,4dione, methyl-3-methoxy-4H-pyran-4-one and hexahydro-3-(methylphenyl)-pyrrolo[1,2a]pyrazine-1,4-dione)	Jaramillo et al., 2020
16.	*Fusarium solani, Alternaria alternate*, and *Neofusicoccum* sp.	*Eupatorium buniifolium*	Essential oil	Limonene, pinene, and sabinene	Cecati et al., 2018
17.	Eleven endophytic fungal strains	*Praxelis clematidea*	Monoterpenoids: (+)-limonene, (-)-limonene, and mycrene	L-limonene -1,2-diol, limonene and linalool oxide	Yang and Wang, 2021
18.	*Aspergillus fumigatus, Rhizopus* sp., *Penicillium radicum*, and *Fusarium proliferatum*	*Lactuca sativa*	Toxic hexavalent form of chromium	Less toxic trivalent form of chromium	Hussain et al., 2018
19.	*Stemphylium lycopersici* and *Sordaria* sp.		Monoacylglycerols	Solketal 1-monoacylglycerol	Rocha et al., 2020
20.	*Penicillium oxalicum*	*Artemisia annua* L.	Triclosan	2-chlorohydroquinone, 2, 4-dichloropheno, and hydroquinone	Tian et al., 2018
21.	*Preussia minima*	*Cupressus lusitanica*	Diterpenes sandaracopimaric and *ent*-isopimaric acids	7β-hydroxy-*ent*-pimara-8(14)-15-dien-19-oic acid, novel diterpenes 7-oxo-8 β-hydroxy-*ent*-pimara-8(14)-15-dien-19-oic, 7-oxo-9β-hydroxy-*ent*-pimara-8(14)-15-dien-19-oic acids, 11α-hydroxyisopimara-8(14)-15-dien-18-oic acid, 7β,11α-dihydroxyisopimara-8(14)-15-dien-18-oic acid, 1β,11α-dihydroxyisopimara-8(14)-15-dien-18-oic acid, and 7β-hydroxyisopimara-8(14)-15-dien-18-oic acid	Ud Din et al., 2018
22.	*Aspergillus* sp. A31,*Curvularia* geniculata P1,*Lindgomycetaceae* P87, and *Westerdykella* sp. P71	*–*	Mercury	Bioremediation of mercury and improved *Aeschynomene fluminensis* and *Zea mays* tolerance to mercury	Pietro-Souza et al., 2020

## Concluding Remarks

This review highlights the potential of fungal endophytes as biocatalysts in selectively designing and synthesizing fundamentally new molecular structures. Such microbial bioconversions serve as attractive alternatives to chemical synthesis under eco-friendly conditions. The confluence of endophytic fungal ecology with its broad bioactive metabolic profile has fringe the start of much-awaited novel biocatalyst discoveries. These endosymbionts of plants have achieved considerable prominence for fulfilling the increasing demand of strategic pure compounds. For this, the utilization of multi-enzyme systems hidden in endophytic community has refined the synthetic space of organic synthesis. Microbial cascade biotransformations with improved retention time, stability, reusability, and enantiomeric purity totally amalgamate with the principles of green chemistry. Furthermore, improvements in recombinant DNA technology, an in-depth study of enzymatic reaction mechanisms, field trials, and high-end sequencing technologies will enhance the conversion performance and applicability of biocatalyst for uncovering many new active pharmacophore molecules.

## Author Contributions

All authors listed have made substantial, direct and intellectual contribution to the work, and approved it for publication.

## Conflict of Interest

The authors declare that the research was conducted in the absence of any commercial or financial relationships that could be construed as a potential conflict of interest.
